# Prophylactic Intra-Arterial Injection of Vasodilator for Asymptomatic Vasospasm Converts the Patient to Symptomatic Vasospasm due to Severe Microcirculatory Imbalance

**DOI:** 10.1155/2014/382484

**Published:** 2014-04-16

**Authors:** Norihito Shimamura, Masato Naraoka, Naoya Matsuda, Kiyohide Kakuta, Hiroki Ohkuma

**Affiliations:** Department of Neurosurgery, Hirosaki University School of Medicine, 5-Zaihuchou, Hirosaki, Aomori Prefecture 036-8562, Japan

## Abstract

*Object*. The strategy to treat asymptomatic angiographic vasospasm following subarachnoid hemorrhage (SAH) is controversial. In this study we review our consecutive vasospasm series and discuss an adequate treatment strategy for asymptomatic vasospasm. *Methods*. From January 2007 to December 2012 we treated 281 aneurysmal SAH cases, with postoperative angiography performed 9 ± 2 days after the onset of SAH. Four asymptomatic cases received intra-arterial (IA) injection of vasodilator due to angiographic vasospasm. All cases improved vasospasm immediately following intervention. But all cases turned symptomatic within 48 hours. We retrospectively analyzed the time-density angiography curve and calculated the time to peak (TTP), mean transit time (MTT), and relative blood flow (rBF). Relative blood flow was calculated as follows. The integration of the value of the time-density curve for the artery was divided by the same value for the internal carotid artery multiplied by the MTT. *Results*. The decrease in TTP and MTT for the etiologic artery was similar to that of the nonetiologic artery. But the improvement in rBF for the etiologic artery and nonetiologic artery was 10% and 17%, respectively. Blood supply to the spastic artery decreased due to iatrogenic steal. *Conclusion*. Prophylactic IA injection of vasodilator in cases of asymptomatic vasospasm can produce symptomatic vasospasm.

## 1. Introduction

Aneurysmal subarachnoid hemorrhage (SAH) impacts approximately 10–21 in 100,000 individuals annually [1–3]. In an aging society, the rupture rate for aged persons is relatively high and can be expected to increase [4–6]. Overall mortality and morbidity are greater than 50% [4, 7, 8]. A major influence on the outcome of postoperated SAH patients is cerebral vasospasm (CVS) [6, 9, 10]. Many factors contribute to the development of CVS: distal microcirculatory failure, poor collateral anatomy, genetic or physiological variations, cortical spread of depolarization, and remodeling [11]. Even when aggressive treatment of CVS was initiated, the reported incidence of symptomatic CVS still ranged from 10 to 50% [6, 12–15]. Intra-arterial (IA) injection of papaverine, nimodipine, nicardipine, fasudil, and milrinone dilates the CVS artery [16–20]. Such IA therapies are effective for symptomatic cases. Prophylactic transluminal balloon angioplasty within 96 hours after SAH also significantly reduces the need for therapeutic angioplasty, but this does not improve poor patient outcome [21].

Recent advances in the noninvasive investigation of cerebral blood flow have led to improved detection of the progression of CVS [22, 23]. The gold standard for the detection of CVS is cerebral digital subtraction angiography (DSA), because this modality measures cerebral circulation time and results in prompt intervention for CVS [24]. Ohkuma et al. detected that peripheral cerebral circulation time was negatively correlated with regional cerebral blood flow using DSA and single photon emission CT in cases of SAH [25]. But the strategy to treat asymptomatic angiographic vasospasm is controversial. We monitored four consecutive asymptomatic cases of angiographic vasospasm that changed to symptomatic CVS within 48 hours after the IA injection of vasodilator. In this study, we analyze the cerebral blood flow in those cases and discuss an adequate treatment strategy for asymptomatic CVS.

## 2. Material and Methods

This study was approved by the Hirosaki University Ethics Committee and we acquired written, informed consent for this study from patients and/or family.

From January 2007 to December 2012 we treated 281 cases of SAH within 72 hours after aneurysm rupture. We maintained normovolemia, along with administering intravenous fasudil hydrochloride. Routine postoperative angiography was carried out 9 ± 2 days after the onset of SAH.

**Figure 1:**
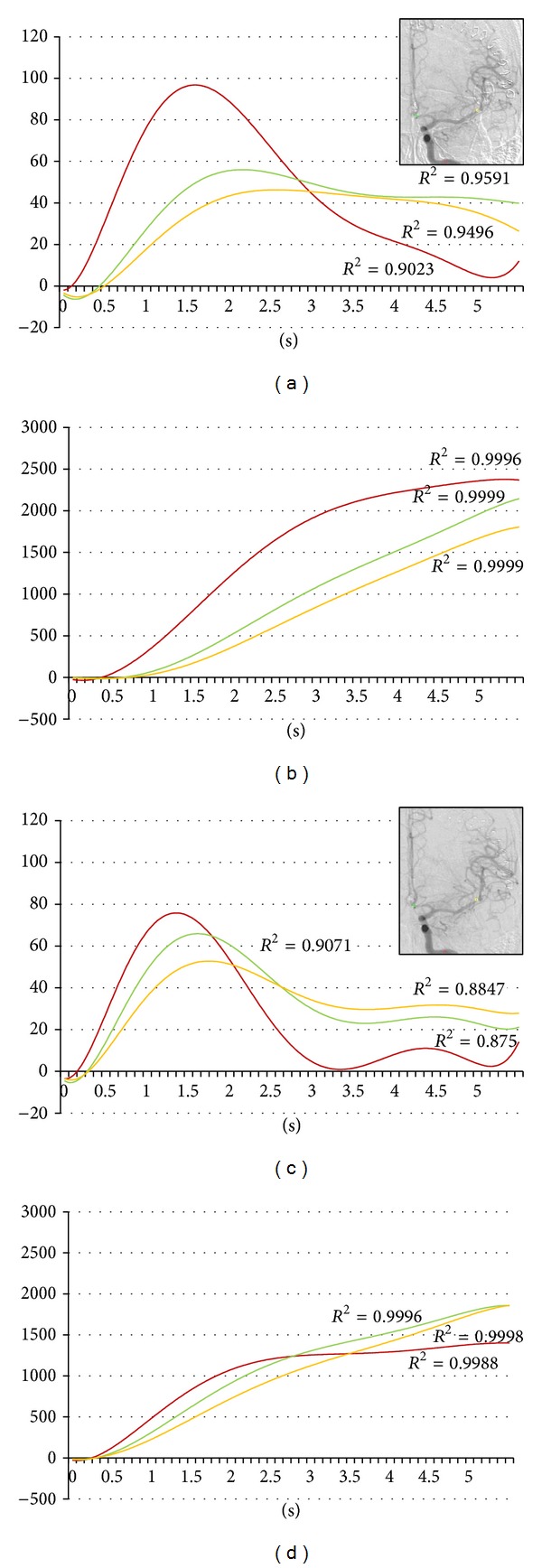
Forty-five-year-old female. Postruptured anterior communicating artery aneurysm clipping. Angiography was done on the ninth day after the subarachnoid hemorrhage. Red, yellow, and green indicate the internal carotid artery, the middle cerebral artery, and the anterior cerebral artery, respectively. (a) Time-density curve for contrast medium for preintra-arterial injection of vasodilator. (b) Integration curve for contrast medium for preintra-arterial injection of vasodilator. (c) Time-density curve for contrast medium for postintra-arterial injection of vasodilator. (d) Integration curve for contrast medium for postintra-arterial injection of vasodilator.

**Figure 2:**
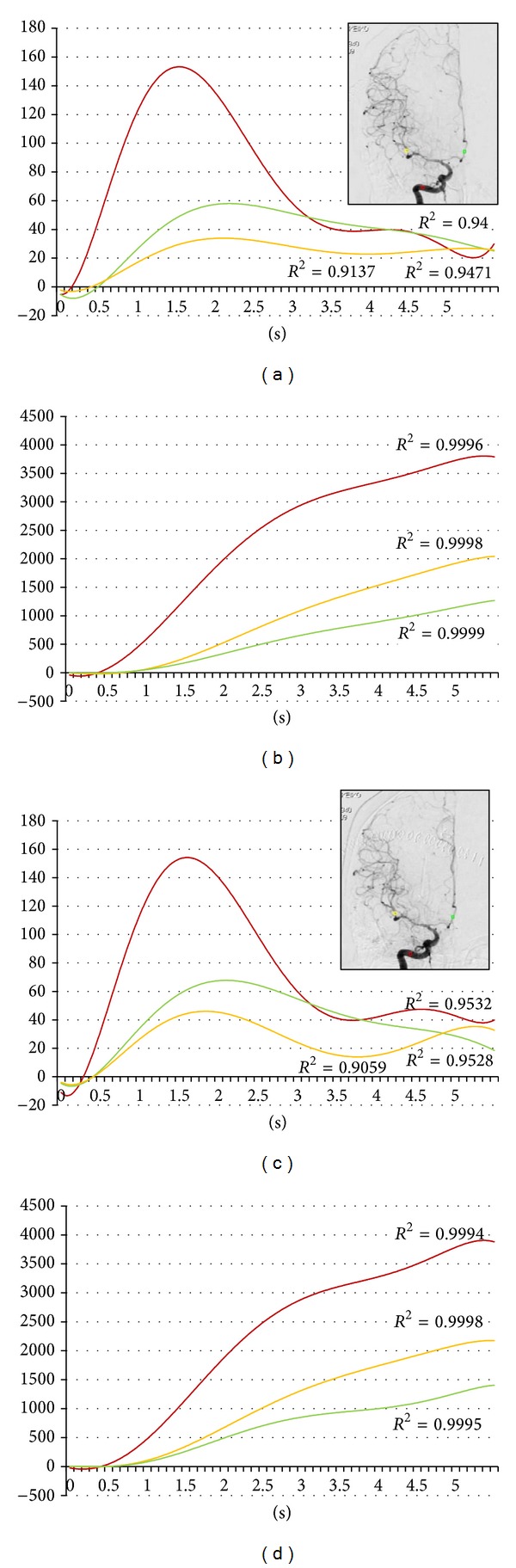
Sixty-nine-year-old female. Postruptured right internal carotid artery aneurysm clipping. Angiography was done on the seventh day after the subarachnoid hemorrhage. Red, yellow, and green indicate the internal carotid artery, the middle cerebral artery, and the anterior cerebral artery, respectively. (a) Time-density curve for contrast medium for preintra-arterial injection of vasodilator. (b) Integration curve for contrast medium for the preintra-arterial injection of vasodilator. (c) Time-density curve for contrast medium for the postintra-arterial injection of vasodilator. (d) Integration curve for contrast medium for the postintra-arterial injection of vasodilator.

**Figure 3:**
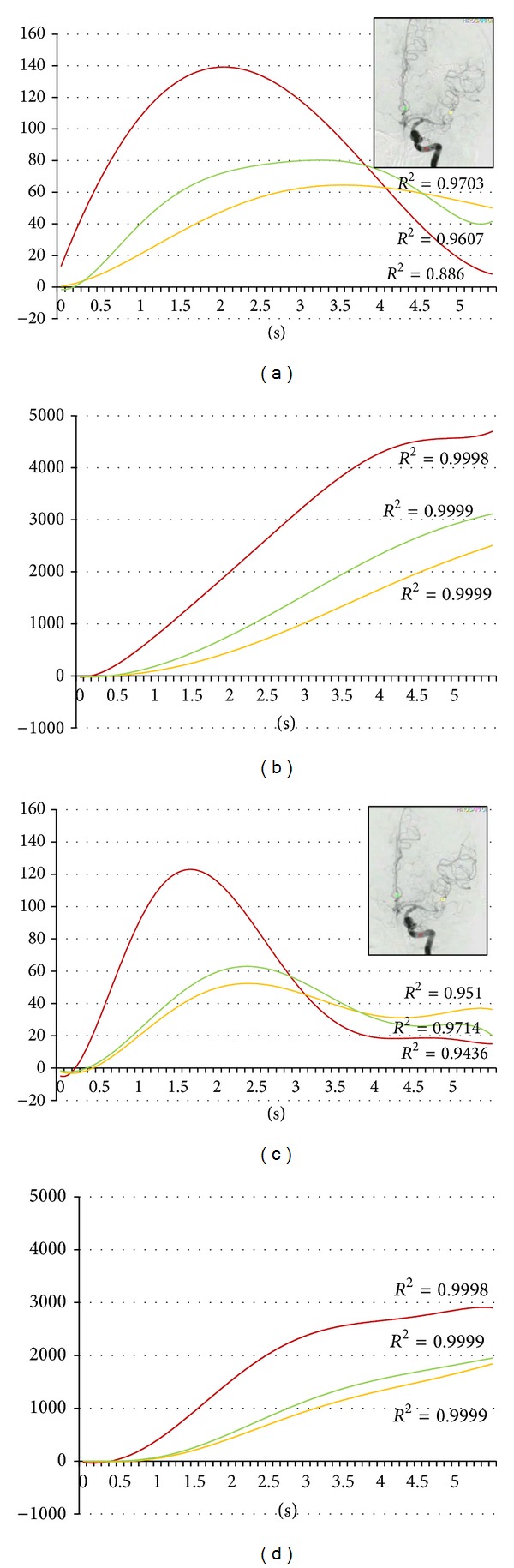
Sixty-nine-year-old female. Postruptured left middle cerebral artery aneurysm clipping. Angiography was done on the ninth day after the subarachnoid hemorrhage. Red, yellow, and green indicate the internal carotid artery, the middle cerebral artery, and the anterior cerebral artery, respectively. (a) Time-density curve for contrast medium for the preintra-arterial injection of vasodilator. (b) Integration curve for contrast medium for the preintra-arterial injection of vasodilator. (c) Time-density curve for contrast medium for the postintra-arterial injection of vasodilator. (d) Integration curve for contrast medium for the postintra-arterial injection of vasodilator.

**Figure 4:**
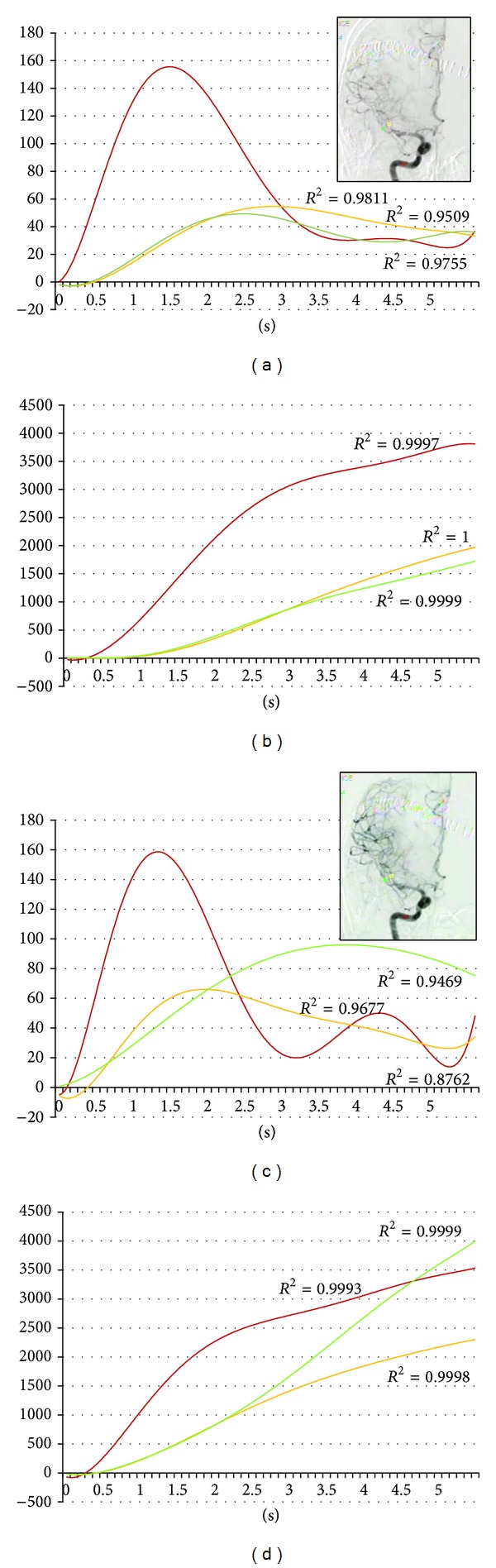
Seventy-five-year-old female. Postruptured right middle cerebral artery aneurysm clipping. Angiography was done on the eighth day after the subarachnoid hemorrhage. Red, yellow, and green indicate the internal carotid artery, the middle cerebral artery, and the anterior cerebral artery, respectively. (a) Time-density curve for contrast medium for the preintra-arterial injection of vasodilator. (b) Integration curve for contrast medium for the preintra-arterial injection of vasodilator. (c) Time-density curve for contrast medium for the postintra-arterial injection of vasodilator. (d) Integration curve for contrast medium for the postintra-arterial injection of vasodilator.

During the first year of this study, we undertook 51 IA therapies in 40 patients. Four asymptomatic vasospasm cases are included in this series (Table 1). Pulsatile IA injection of 30 mg fasudil hydrochloride and vasodilators was applied to spastic arteries. All four cases improved vasospasm immediately following intervention (Figures 1, 2, 3, and 4). But all cases changed to symptomatic CVS within 48 hours. We subsequently analyzed the parameters of cerebral blood flow to clarify these unexpected events.

Digital subtraction angiography (DSA) was performed by a certified neurosurgeon. A 4Fr or 6Fr catheter was inserted via the femoral artery into each internal carotid artery, and its tip was set at the level of the second cervical vertebra. Six milliliters of contrast agent was injected into the internal carotid artery at 4 mL/s by autoinjector. Images were obtained at a rate of six frames per second with the use of a DSA unit (Artis dBA Twins, Siemens, Germany) with a pixel matrix of 1024 × 1024, and the DSA images were stored in the computer system.

The regions of interest (ROI) were set in the vertical petrous portion of the internal carotid artery (red), the etiologic artery (yellow), and the nonetiologic artery (green) on the images of an anteroposterior projection (Figures 1–4) with avoidance of veins. The time-density curve and integration curve of the contrast media in each ROI were obtained using a U11437 luminance analyzer (Hamamatsu Photonics, Shizuoka, Japan) from the series of DSA images. The initial time was defined as the time at which each curve took an upward turn in the ROI of C5, and the entire first-pass curve was established during 6 seconds [25–27]. The time-density curve and the integration curve were fitted to polynomial approximation, and a coefficient of determination (*R* ^2^) that was over 0.8 was accepted [28].

The time to peak opacification was defined as time to peak (TTP). The mean transit time (MTT) in each ROI was determined as ∑(0–*∞*)*Ct*/∑(0–*∞*)*C*, where *C* is the quantity of contrast medium remaining at the site and *t* is the time after the contrast media is injected. The time to half peak of the integration curve was defined as the MTT [29]. Relative blood flow (rBF) was calculated as

(1) ∑(0–∞)Crt/{∑(0–∞)Crt/∑(0–∞)Cr}{∑(0–∞)Ctic/∑(0–∞)Cic}  =∑(0–∞)Cr∗∑(0–∞)Cic/∑(0–∞)Ctic,

where “*r*” is the region of the artery and “ic” is the internal carotid artery [30].

## 3. Results 

**Figure 5:**
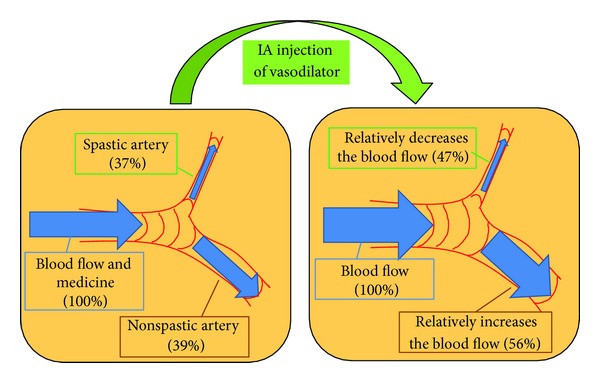
Mechanism of iatrogenic blood steal in a case of asymptomatic vasospasm. Vasodilator easily flows into nonspastic arteries; these nonetiologic arteries then advanced a much greater supply of blood. The improvement in blood flow of the spastic artery is relatively smaller than that of the nonspastic artery (compared to the original).

The decreases in TTP of the internal carotid artery, nonetiologic artery, and etiologic artery were 0.05 seconds, 0.60 seconds, and 0.73 seconds, respectively (Table 2). The decreases in MTT of the internal carotid artery, nonetiologic artery, and etiologic artery were 0.28 seconds, 0.38 seconds, and 0.55 seconds, respectively (Table 2). But improvements in rBF of the nonetiologic artery and the etiologic artery were 17% and 10%, respectively (Table 2). Improvement of blood flow in the nonetiologic artery was superior to the etiologic artery (Figure 5).

## 4. Discussion

We first detected iatrogenic blood flow steal after the IA injection of vasodilator for asymptomatic CVS. It was considered possible that vasodilator flowed into the nonspastic arteries and that these nonetiologic arteries then advanced a much greater supply of blood (Figure 5). The insufficiency of blood flow in the asymptomatic spastic artery induced symptomatic CVS after treatment. Limited vasodilatory reserve and dysfunctional autoregulation have been observed in patients with SAH, while arterial vasodilation is nonuniform [31]. Tekle et al. reported that eleven of 41 endovascular-treated symptomatic CVS cases suffered a renewed occurrence of ischemic symptoms in previously asymptomatic arterial distribution [32]. In our series, eight symptomatic cases (22.2%) underwent multiple IA administrations of medicine. We speculate that the same steal phenomenon occurred in those cases of multiple treatment and led to symptomatic vasospasm. Iwabuchi et al. reported that IA injection of fasudil hydrochloride reduced time to peak in the CVS artery dose dependently [26]. In their experience, 13 asymptomatic vasospasm cases did not change to symptomatic vasospasm, but the blood flow of spastic arteries was not analyzed.

Hesselink et al. reported that the integrated area of the time-density curve of DSA depended on flow, vessel size, amount of iodine injected, framing rate, and the kVp [33]. Only the flow of the artery is different in each ROI case. A change in the integrated area of the time-density curve thus means a change in blood flow. Moreover, Lois et al. [30] reported that an inverse integration value for the time-density curve of DSA correlated with blood flow. Calculation of relative blood flow depends on the inverse integration value for the time-density curve for DSA. To calculate the relative blood flow of the spastic artery, we used the integration value of the time-density curve for contrast medium in the internal carotid artery. Kruger et al. reported that the time to maximum opacification can be used to determine absolute and relative blood flow using a phantom model [34]. Human studies show that blood pressure and heart rate change continually, correlating with cerebral blood flow. To compare pretreatment flow to posttreatment flow we divided the integration value of the time-density curve for contrast medium by the same value for the internal carotid artery, for purposes of standardization.

Even though we performed an IA injection of vasodilator via the M1 portion of the middle cerebral artery in case 4, the patient changed to symptomatic CVS. To prevent a change from asymptomatic CVS to symptomatic CVS, advanced superselective IA injection should be considered. IA injection of vasodilator is carried out directly to the spastic artery via M2, M3, or A2. But this method is difficult and risky for untrained doctors because of the tiny size of the spastic artery. Therefore, prophylactic IA injection of vasodilator for asymptomatic CVS cases is not beneficial.

## 5. Conclusions

Prophylactic IA injection of vasodilator for asymptomatic CVS cases can produce symptomatic CVS. We do not recommend prophylactic IA of vasodilator for asymptomatic CVS.

## Figures and Tables

**Table 1:** Analyzed patients. All cases are female and the Fisher Group of all cases is three.

	Age	Location of aneurysm	Hunt-Hess grade	Day of postclipping DSA	Position of catheter for IA	Onset of symptom after IA	Etiologic artery	GOS at 30 days
Case 1	45	A. com	2	9	Lt. IC	8 hours	Lt. MCA	Good recovery
Case 2	69	Rt. IC	3	7	Rt. IC	2 days	Rt. MCA	Moderate disability
Case 3	69	Lt. MCA	1	9	Lt. IC	1 day	Lt. MCA	Good recovery
Case 4	75	Rt. MCA	2	8	Rt. MCA	1 day	Upper branch of rt. MCA	Moderate disability

A. com: anterior communicating aneurysm. DSA: digital subtraction angiography. GCS: Glasgow Outcome Scale. IA: intra-arterial injection of medicine. IC: internal carotid artery. MCA: middle cerebral artery.

**Table 2:** Parameters of blood velocity.

	Internal carotid artery	Nonetiologic artery	Etiologic artery
Time to peak (sec)			
Before IA	1.70 ± 0.22	2.65 ± 0.54	2.83 ± 0.61
After IA	1.65 ± 0.31	2.05 ± 0.58	1.9 ± 0.51
Mean transit time (sec)			
Before IA	1.98 ± 0.22	3.08 ± 0.22	3.08 ± 0.15
After IA	1.70 ± 0.37	2.70 ± 0.55	2.53 ± 0.12
Relative blood flow (%)			
Before IA	100	39 ± 13	37 ± 14
After IA	100	56 ± 21	47 ± 18

IA: intra-arterial injection of vasodilator. All values are listed as mean ± standard deviation.
